# Proteome profiling identifies circulating biomarkers associated with hepatic steatosis in subjects with Prader-Willi syndrome

**DOI:** 10.3389/fendo.2023.1254778

**Published:** 2023-11-15

**Authors:** Devis Pascut, Pablo J. Giraudi, Cristina Banfi, Stefania Ghilardi, Claudio Tiribelli, Adele Bondesan, Diana Caroli, Alessandro Minocci, Graziano Grugni, Alessandro Sartorio

**Affiliations:** ^1^ Liver Cancer Unit, Fondazione Italiana Fegato - ONLUS, Trieste, Italy; ^2^ Metabolic Liver Disease Unit, Fondazione Italiana Fegato - ONLUS, Trieste, Italy; ^3^ Unit of Functional Proteomics, Metabolomics, and Network analysis, Centro Cardiologico Monzino, IRCCS, Milan, Italy; ^4^ Istituto Auxologico Italiano, Istituto di Ricovero e Cura a Carattere Scientifico (IRCCS), Experimental Laboratory for Auxo-endocrinological Research, Piancavallo-Verbania, Italy; ^5^ Istituto Auxologico Italiano, Istituto di Ricovero e Cura a Carattere Scientifico (IRCCS), Division of Metabolic Diseases, Piancavallo-Verbania, Italy; ^6^ Istituto Auxologico Italiano, Istituto di Ricovero e Cura a Carattere Scientifico (IRCCS), Division of Auxology, Piancavallo-Verbania, Italy

**Keywords:** proteomics, proteome, circulating biomarkers, PWS, steatosis, cardiovascular, metabolic, MAFLD CDH2

## Abstract

**Introduction:**

Prader-Willi syndrome (PWS) is a rare genetic disorder characterized by loss of expression of paternal chromosome 15q11.2-q13 genes. Individuals with PWS exhibit unique physical, endocrine, and metabolic traits associated with severe obesity. Identifying liver steatosis in PWS is challenging, despite its lower prevalence compared to non-syndromic obesity. Reliable biomarkers are crucial for the early detection and management of this condition associated with the complex metabolic profile and cardiovascular risks in PWS.

**Methods:**

Circulating proteome profiling was conducted in 29 individuals with PWS (15 with steatosis, 14 without) using the Olink Target 96 metabolism and cardiometabolic panels. Correlation analysis was performed to identify the association between protein biomarkes and clinical variables, while the gene enrichment analysis was conducted to identify pathways linked to deregulated proteins. Receiver operating characteristic (ROC) curves assessed the discriminatory power of circulating protein while a logistic regression model evaluated the potential of a combination of protein biomarkers.

**Results:**

CDH2, CTSO, QDPR, CANT1, ALDH1A1, TYMP, ADGRE, KYAT1, MCFD, SEMA3F, THOP1, TXND5, SSC4D, FBP1, and CES1 exhibited a significant differential expression in liver steatosis, with a progressive increase from grade 1 to grade 3. FBP1, CES1, and QDPR showed predominant liver expression. The logistic regression model, -34.19 + 0.85 * QDPR*QDPR + 0.75 * CANT1*TYMP - 0.46 * THOP1*ALDH1A, achieved an AUC of 0.93 (95% CI: 0.63-0.99), with a sensitivity of 93% and specificity of 80% for detecting steatosis in individuals with PWS. These biomarkers showed strong correlations among themselves and were involved in an interconnected network of 62 nodes, related to seven metabolic pathways. They were also significantly associated with cholesterol, LDL, triglycerides, transaminases, HbA1c, FLI, APRI, and HOMA, and showed a negative correlation with HDL levels.

**Conclusion:**

The biomarkers identified in this study offer the potential for improved patient stratification and personalized therapeutic protocols.

## 1 Introduction

Prader-Willi syndrome (PWS) is a rare genetic disorder characterized by various physical, cognitive, and behavioral symptoms. It is caused by the lack of expression of genes located on the paternal chromosome 15q11.2-q13, resulting in various metabolic abnormalities and hormonal dysregulation (1, 2). PWS is associated with severe obesity and is considered the most common syndromic form of life-threatening obesity (3). The excessive weight gain in individuals with PWS is primarily driven by hyperphagic behavior and a dysregulated appetite control mechanism (4, 5).

While obesity is a prominent feature of PWS, the metabolic profile and cardiovascular complications associated with the syndrome differ from those observed in individuals with non-syndromic obesity (6, 7). Individuals with PWS exhibit distinctive fat distribution patterns, with a higher fat mass percentage, particularly in the abdominal, buttocks, and thigh regions, despite having reduced fat-free mass (4, 8). Individuals with PWS often have reduced visceral fat deposits and a predominant accumulation of subcutaneous adipose tissue compared to individuals with common obesity (9). These unique metabolic characteristics contribute to a distinct metabolic phenotype in PWS, with lower insulin levels and higher insulin sensitivity relative to obese individuals without PWS (10). Despite the metabolic advantages associated with PWS, individuals with this syndrome are still at risk for developing various comorbidities, including type 2 diabetes mellitus, metabolic syndrome, hepatobiliary complications, and cardiovascular and respiratory problems (6, 11). Of particular concern is the presence of metabolic-associated fatty liver disease (MAFLD), commonly associated with obesity. Although individuals with PWS have a lower prevalence of severe MAFLD (12, 13), of MAFLD PWS remains challenging. Given the complex metabolic profile and the risk of cardiovascular complications in individuals with PWS, there is a need for reliable biomarkers for the e arly detection and management of these conditions. Protein biomarkers have emerged as potential candidates for detecting and characterizing liver steatosis and more advanced stages of the disease. These proteins are involved in pathways related to lipid transport, storage, and metabolism. They include the fatty acid binding protein 4 (FABP4) (14–16), retinol-binding protein 4 (RBP4) (15, 17), and adiponectin (18, 19), which have been associated with liver steatosis and insulin resistance. Elevated ALT and AST levels are often observed in individuals with liver steatosis (20, 21). Recently, several proteomics studies have identified novel plasma protein candidates, including a panel of five proteins (PIGR, DPP4, ANPEP, TGFB1, and APOE) correlating with the liver injury (22). Additional candidates have also been combined in diagnostic models, such as the logistic regression model comprising four proteins (ADAMTSL2, AKR1B10, CFHR4, and TREM2), BMI, and/or type 2 diabetes mellitus status, to identify individuals at-risk of steatohepatitis (23) and/or advanced fibrosis (24). However, the applicability of these markers for MAFLD and advanced stages of the disease in PWS subjects requires further validation. Multiple omics approaches may expand the pool of biomarker candidates for different stages of MAFLD and metabolic complications in this rare disease, supporting clinicians and researchers in understanding the cardio-metabolic status and hepatic health of individuals with PWS, facilitating early detection, monitoring, and management of liver-related complications. Ultimately, this knowledge can lead to improved therapeutic strategies and outcomes for individuals with PWS.

## 2 Materials and methods

### 2.1 Patients

Thirty-one individuals with PWS (15 females, 16 males, mean age ± SD: 31.94 ± 12.2 years), hospitalized for a three-week multidisciplinary body weight reduction program at the Division of Auxology, Istituto Auxologico Italiano, IRCCS, Piancavallo-Verbania, Italy, were recruited for the present study. All subjects with PWS showed the typical clinical phenotype (2). Twenty-seven subjects had an interstitial deletion of the long proximal arm of chromosome 15 (del15q11-q13), while 4 patients had uniparental maternal disomy for chromosome 15. Twenty-two patients were classified as obese (Body mass index (BMI) > 30 kg/m2), while the remaining 9 had a BMI lower than 30. BMI ranged between 20 and 55.2 kg/m2 (mean ± SD: 37.0 ± 9.1 kg/m2). Ten patients were treated with recombinant human growth hormone (rhGH) at the time of the study and 4 were previously treated during adulthood. Nine of these and 9 other patients had been treated at pediatric age. Eight patients had never received GH therapy. Patients received rhGH treatment for a period ranging between 2 and 17 years. Liver steatosis was assessed through liver ultrasonography by an experienced echographist (AM) according to standardized criteria (25, 26), as previously described (27). The Ethics Committee of Istituto Auxologico Italiano Milan, Italy (ethical committee code: CE: 2022_03_15_07; research code: 01C216; acronym: PROTEOMARKER) approved the study. All procedures in the study complied with the Helsinki Declaration of 1975, as revised in 2008. The research procedure was explained to each participant and written informed consent was obtained by subjects and their parents, when it was appropriate.

### 2.2 Serum collection

After an overnight fast, blood samples were obtained through standard venipuncture using BD Vacutainer^®^ serum separating tubes (BD - Plymouth PL6 7BP, UK). The tubes were then centrifuged at 1900 x g for 10 minutes at 4°C. Following the initial centrifugation, the resulting supernatants were carefully transferred into new tubes. Subsequently, samples were centrifug ed at 16000 x g for 10 minutes at 4°C. Finally, the supernatants were aliquoted into new tubes and promptly frozen at -80°C to ensure long-term storage until further analysis.

### 2.3 Circulating proteome profiling and analysis

Blood samples were analyzed using the proximity extension assay (PEA) on Olink Target 96 metabolism and cardiometabolic panels (Olink Proteomics, Uppsala, Sweden). In this process, the target proteins specifically bind to double oligonucleotide-labeled antibody probes. Subsequently, microfluidic real-time PCR amplifies the oligonucleotide sequence for quantitative DNA sequence detection (28). Threshold cycle (Ct) data resulting from internal and external controls were subjected to quality control and normalization. Protein levels were measured on a relative scale and presented as a normalized protein expression (NPX) unit, represented on a log2 scale. Higher NPX values correspond to a high protein concentration. A list of proteins included in the analysis is shown in **Table S1**. Data visualization, exploration, and initial statistical analysis were conducted using the Olink Statistical Analysis web-based app. The NPX dataset was uploaded into the application, and samples that did not meet quality controls were excluded from the analysis. The samples were then categorized based on the grade of steatosis, and an analysis of variance (ANOVA) was performed on each assay. The results were presented as NPX median values and the inter-quartile range (IQR) for each marker within the sample group, unless differently specified. The reported p-values from the ANOVA analysis were adjusted for multiple testing using the Benjamini-Hochberg method.

### 2.4 Bioinformatics analysis

To identify relevant pathways and perform enrichment analyses, we utilized various databases including Gene Ontology (GO) (http://geneontology.org/), Kyoto Encyclopedia of Genes and Genomes (KEGG) (https://www.genome.jp/kegg/pathway.html), Reactome (https://reactome.org/), and GeneCards (https://www.genecards.org/). During the enrichment analyses, we associated all the significantly expressed proteins with their respective terms or pathways in the GO or KEGG database. The analysis was carried out using the hypergeometric test or Fisher’s exact test with default settings. This process allowed us to gain insights into the functional implications of the identified proteins in the context of liver steatosis. In addition, the expression data of the proteins of interest were investigated in the GTEx data portal (http://gtexportal.org). By querying the selected tissue expression database, a heatmap with pseudocolor representation was generated. This is to visualize the expression patterns of these proteins across different tissues. STRING Version 11.5 (https://string-db.org) was employed to construct a protein-protein interaction network. The network was generated by using a query of five proteins, as described in the results section.

### 2.5 Correlation analysis

Correlations between variables were investigated using the Spearman rank correlation test (considering the low sample size). Correlation matrices were generated with R 4.2.2 (29) by using the Hmisc version 5.0-1 (30, 31), Performance Analytics version 2.0.4 (32), and Corrplot version 0.92 (33) packages.

### 2.6 Bootstrap analysis

We assessed the robustness of the logistic regression model using a non-parametric bootstrap simulation with out-of-bag prediction over 10,000 iterations. The corresponding R script can be found in **Supplementary File 1**.

### 2.7 Statistical methods

T-test was used to determine significant differences among normally distributed continuous variables, while the Mann-Whitney was used for non-normally distributed variables. The Chi-Squares test was used for categorical variables. The Kruskal-Wallis test in the One-Way ANOVA procedure with Benjamini Hochberg correction was used for multiple comparisons. The receiver operating characteristic (ROC) curves were plotted to estimate the discriminatory potential of the circulating protein biomarkers. A hierarchical forward selection with switching one-way logistic analysis was used to estimate the discriminatory potential of the protein biomarker combination. Analyses were performed using NCSS 11 Software (2016) (NCSS, LLC. Kaysville, Utah, USA, ncss.com/software/ncss).

## 3 Results

### 3.1 Characteristics of the participants

Olink proteomic analysis of individuals with PWS was performed to identify circulating biomarkers associated with the presence of steatosis. The studied population was grouped according to absence (S0) or presence (S1-S2-S3) of steatosis. **Table 1** shows the clinical characteristics of the participants. Significant differences (p < 0.05) between groups were observed for BMI, diastolic pressure, insulin, HOMA index, glycated hemoglobin (HbA1c), C-reactive protein, triglycerides, fatty liver index – FLI, and the presence of metabolic syndrome. All these parameters were higher in the group with steatosis.

**Table 1:** Clinical characteristics of the studied population.

Variable	Prader-Willi cohort (n=31)		*p-value*
	S0; n=16	S1-S2-S3; n= 15	S1-S2-S3 vs S0
Age (years)	32 ± 11	32 ± 13	0.82
Gender-female (n, %)	8, 50%	8, 53%	0.85
BMI (kg/m^2^)	33 ± 8	41 ± 8	**0.025**
Waist circumference (cm)	110 ± 19	123 ± 18	0.07
50 Hz (ohm)	623 ± 77	571 ± 152	0.27
FFM (kg)	48 ± 10	47 ± 7	0.67
FFM (%)	57 ± 10	50 ± 7	0.06
mREE (kcal)	1695 ± 304	1655 ± 245	0.73
Systolic pressure (mm Hg)	124 ± 6	129 ± 12	0.18
Diastolic pressure (mm Hg)	77 ± 4	82 ± 5	**0.005**
Fasting glucose (mg/dL)	86 ± 10	121 ± 75	0.09
Insulin (mU/L)	9 ± 3	15 ± 7	**0.012**
HOMA-IR	1.8 ± 0.7	4.2 ± 2.2	**0.001**
HbA1c (%)	5.4 ± 0.4	7.0 ± 2.4	**0.018**
Total cholesterol (mg/dL)	174 ± 31	189 ± 51	0.35
HDL cholesterol (mg/dL)	54 ± 18	44 ± 11	0.075
LDL cholesterol (mg/dL)	110 ± 25	120 ± 38	0.4
Triglycerides (mg/dL)	98 ± 38	200 ± 165	**0.03**
c-reactive protein (mg/dL)	0.33 ± 0.40	1.20 ± 0.72	**0.003**
AST (U.I./L)	22 ± 9	28 ± 16	0.21
ALT (U.I./L)	33 ± 41	41 ± 35	0.6
GGT (U.I./L)	20 ± 14	69 ± 103	0.09
Fatty Liver Index - FLI	61 ± 35	93 ± 13	**0.003**
APRI	0.27 ± 0.1	0.37 ± 0.2	0.2
FIB-4	0.63 ± 0.4	0.61 ± 0.4	0.9
MetS yes (%)	2 (12%)	9 (60%)	**0.006**

BMI, body mass index; FFM, Fat-free mass; mREE, measures resting energy expenditure; HOMA-IR, homeostatic model assessment-insulin resistance; HbA1c, glycated hemoglobin; HDL, high-density lipoprotein; LDL, low-density lipoprotein; ALT, alanine aminotransferase; AST, aspartate aminotransferase; GGT, gamma-glutamyl transferase; MetS, metabolic syndrome, Steatosis grade 0 S0, Steatosis grade 1, 2 and 3 S1-S2-S3. p<0.05 was considered statistically significant vs. respective controls, S0 group was used as control. Data are shown as mean ± SD for continuous variables, and number (%) for binary variables. t-test was used to test for significant differences within continuous variables that were normally distributed, while Mann-Whitney and Kruskal-Wallis when non-normally distributed. Chi-Squares test was used for categorical variables. In bold are indicated the significant p-values.

### 3.2 Sex differences in the circulating proteome of individuals with PWS

**Figure 1:**
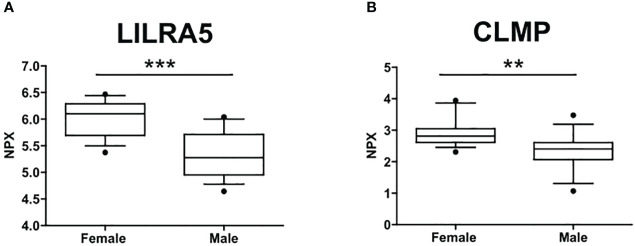
Sex differences in the circulating protein biomarkers. **(A)** LILRA5 showed a median NPX expression of 6.1 (5.7-6.29) in females and a median NPX expression of 5.28 (4.95-5.71) in males. **(B)** CLMP showed a median NPX expression of 2.8 (2.61-3.05) in females and a median NPX expression of 2.4 (2.07-2.61) in males. Values are presented as median with their respective interquartile ranges [IQR, 25th-75th percentile]. Statistical significance levels are denoted as follows: **, p<0.01; ***, p<0.001.

The circulating proteome was investigated using the Olink metabolism and cardiometabolic panels, each consisting of 92 different human protein biomarkers (**Table S1**). Two samples were removed from subsequent analysis due to alterations identified in the Olink internal quality control test. Two proteins, Leukocyte Immunoglobulin Like Receptor A5 (LILRA5) and CXADR Like Membrane Protein (CLMP) (from the metabolic panel), exhibited differential expression between males (n=15) and females (n=14) (p=0.046 and p=0.049, respectively). Notably, both biomarkers showed downregulation in males, with a median expression of 5.28 compared to 6.1 for LILRA5 and 2.4 compared to 2.8 for CLMP (**Figure 1**).

### 3.3 Circulating protein biomarkers associated with the presence of liver steatosis in individuals with PWS

**Figure 2:**
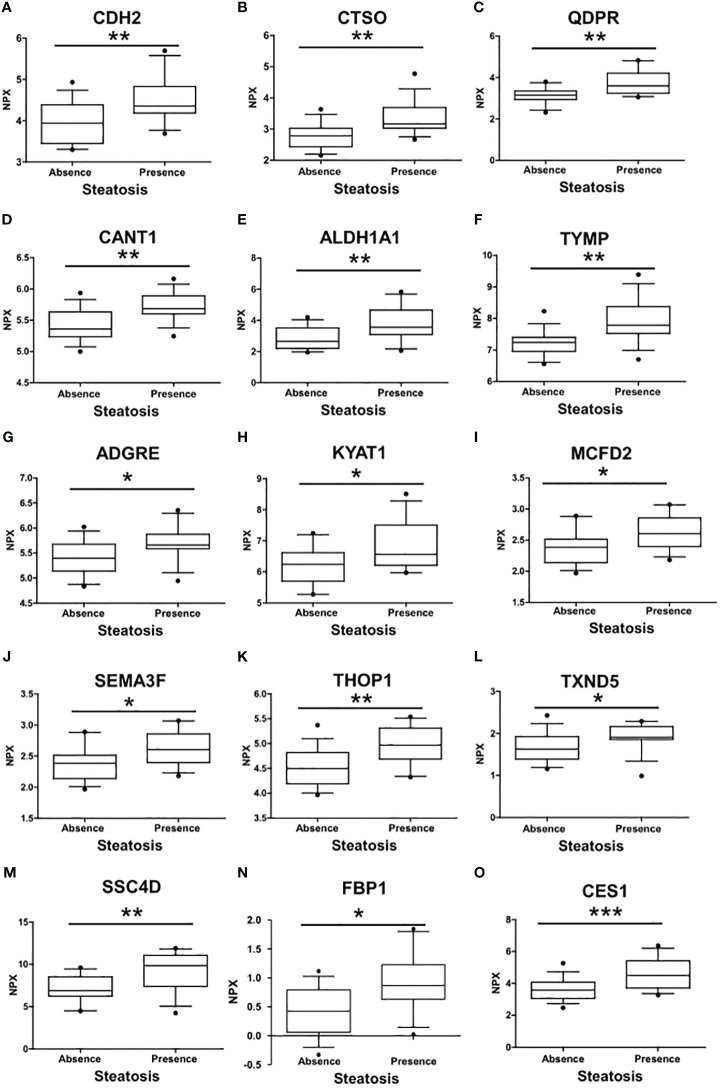
Differences in the circulating protein biomarkers according to the presence of liver steatosis. The analysis was performed by comparing individuals with PWS without steatosis and individuals with PWS with steatosis (including steatosis grade 1, grade 2, and 3). Values are presented as median with their respective interquartile ranges [IQR, 25th-75th percentile]. Statistical significance levels are denoted as follows: *, p<0.05; **, p<0.01; ***, p<0.001.

A comprehensive analysis revealed that a total of 15 proteins exhibited significant differential expression according to the presence of liver steatosis (**Figure 2**, **Table 2**). These include Cadherin 2 (CDH2), Cathepsin O (CTSO), Quinoid Dihydropteridine Reductase (QDPR), Calcium Activated Nucleotidase 1 (CANT1), Aldehyde Dehydrogenase 1 Family Member A1 (ALDH1A1), Thymidine Phosphorylase (TYMP), Adhesion G Protein-Coupled Receptor E2 (ADGRE), Kynurenine Aminotransferase 1 (KYAT1), Multiple Coagulation Factor Deficiency 2, ER Cargo Receptor Complex Subunit (MCFD2), Semaphorin 3F (SEMA3F), Thimet Oligopeptidase 1 (THOP1), Thioredoxin Domain Containing 5 (TXND5), Scavenger Receptor Cysteine Rich Family Member With 4 Domains (SSC4D), Fructose-Bisphosphatase 1 (FBP1), and Carboxylesterase 1 (CES1). Fourteen biomarkers belong to the OLINK metabolism panel, while only CES1 belongs to the OLINK cardiometabolic panel. All these circulating proteins showed higher expression levels in subjects with steatosis. Upon investigation in the GTEx portal (https://gtexportal.org/home/), we found that the liver exhibited the highest mRNA expression levels of FBP1, CES1, and QDPR among the analyzed organs (**Figure S1**) suggesting their role in liver function. Indeed, those candidates map on several liver-related pathways (**Table S2**). FBP1 is involved in several pathways including the Angiopoietin-like protein 8 regulatory pathway, disorders of the fructose metabolism pathway, and glycolysis and gluconeogenesis pathways. CES1 is implicated in cholesterol and sphingolipid transport-related pathways, as well as in the transport of molecules from the Golgi apparatus and endoplasmic reticulum to the apical membrane. CES1 also participates in lipid metabolic processes, including cholesterol biosynthesis and the detoxification response to toxic substances. QDPR is associated with pathways involved in folate metabolism, L-phenylalanine degradation I (aerobic), and phenylalanine degradation/tyrosine biosynthesis. These pathways are relevant for the breakdown of specific amino acids and the utilization of folate in various metabolic processes.

**Table 2:** Protein biomarkers associated with the presence of liver steatosis.

Assay	UniProt	Panel	Prader-Willi cohort (n=29)		p-value
			S0; n=14	S1-S2-S3; n= 15	S1-S2-S3 vs S0
**CDH2**	P19022	Olink Metabolism	3.94 (3.45 - 4.38)	4.35 (4.20 - 4.83)	0.005
**CTSO**	P43234	Olink Metabolism	2.78 (2.43 - 3.03)	3.17 (3.03 - 3.69)	0.003
**QDPR**	P09417	Olink Metabolism	3.14 (2.93 - 3.35)	3.60 (3.24 - 4.21)	0.002
**CANT1**	Q8WVQ1	Olink Metabolism	5.36 (5.24 - 5.63)	5.68 (5.60 - 5.90)	0.003
**ALDH1A1**	P00352	Olink Metabolism	2.66 (2.20 - 3.52)	3.56 (3.10 - 4.66)	0.008
**TYMP**	P19971	Olink Metabolism	7.24 (6.95 - 7.40)	7.78 (7.53 - 8.37)	0.002
**ADGRE2**	Q9UHX3	Olink Metabolism	5.40 (5.14 - 5.68)	5.66 (5.59 - 5.90)	0.036
**KYAT1**	Q16773	Olink Metabolism	6.24 (5.70 - 6.62)	6.57 (6.21 - 7.51)	0.015
**MCFD2**	Q8NI22	Olink Metabolism	2.38 (2.14 - 2.51)	2.60 (2.40 - 2.86)	0.020
**SEMA3F**	Q13275	Olink Metabolism	4.05 (3.80 - 4.20)	4.22 (4.10 - 4.83)	0.017
**THOP1**	P52888	Olink Metabolism	4.50 (4.20 - 4.82)	4.97 (4.69 - 5.31)	0.005
**TXNDC5**	Q8NBS9	Olink Metabolism	1.63 (1.39 - 1.93)	1.91 (1.86 - 2.16)	0.037
**SSC4D**	Q8WTU2	Olink Metabolism	6.89 (6.25 - 8.53)	9.86 (7.39 - 11.10)	0.009
**FBP1**	P09467	Olink Metabolism	0.43 (0.06 - 0.79)	0.87 (0.63 - 1.23)	0.014
**CES1**	P23141	Olink cardiometabolic	3.55 (3.10 - 3.98)	4.82 (3.71 - 5.42)	0.001

Expression values are reported as NPX median value (IQR). S0, Steatosis grade 0; S1, Steatosis grade 1; S2, Steatosis grade 2; S3, Steatosis grade 3.

### 3.4 The analysis of circulating proteome identifies several biomarkers associated with different grades of liver steatosis

**Figure 3:**
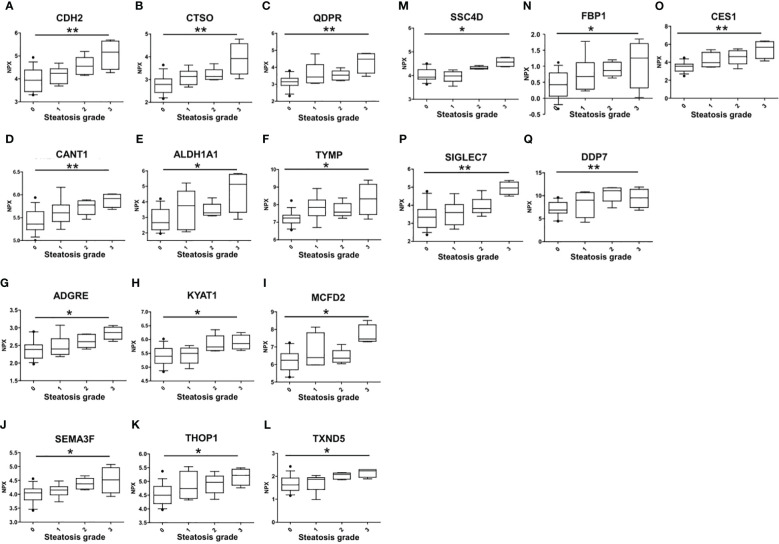
Differences in the circulating protein biomarkers according to the different grades of liver steatosis. The comparison was performed by considering separately the groups with different grades of liver steatosis (S0 = 14, S1 = 6, S2 = 5, S3 = 4). Values are presented as median with their respective interquartile ranges [IQR, 25th-75th percentile]. Statistical significance levels are denoted as follows: *, p<0.05; **, p<0.01.

The association of circulating protein biomarkers from the two selected OLINK panels was explored is subjects with different stages of steatosis. The analysis confirmed the association of the 15 candidates with the presence of steatosis and showed a progressive increase with the degree of steatosis (**Table 3**, **Figure 3**). Two circulating proteins Sialic Acid Binding Ig-Like Lectin 7 (SIGLETC7) and Dipeptidyl Peptidase 7 (DPP7), also showed significant differences according to the degree of steatosis (**Table 3**, **Figures 3P, Q**). In particular, SIGLEC7 was higher in the S2-S3 groups compared to the S0-S1 groups (**Table 3**). On the contrary, ALDH1A and TYMP demonstrated different patterns (**Figures 3E, F**) as they initially increased from S0 to S1, followed by a slight decrease at S2, and then increase again at S3. The SSC4D exhibited a progressive increase from S0 to S2, indicating a correlation with the severity of liver steatosis. The expression level remained higher at S3 than that observed at S0 and S2 (**Figure 3M**).

**Table 3:** Protein biomarkers significantly associated with the different grades of liver steatosis.

Assay	UniProt	Steatosis Grade				meansq	Adj_pval
		S0	S1	S2	S3		
**CDH2**	P19022	3.94 (3.44-4.38)	4.24 (3.79-4.43)	4.55 (4.19-4.93)	5.16 (4.41-5.64)	4.6	0.005
**CTSO**	P43234	2.78 (2.43-3.03)	3.13 (2.78-3.36)	3.13 (3.00-3.43)	3.92 (3.24-4.58)	3.9	0.005
**QDPR**	P09417	3.14 (2.93-3.35)	3.42 (3.08-4.16)	3.54 (3.78-3.25)	4.48 (3.66-4.80)	4.1	0.007
**SIGLEC7**	Q9Y286	3.93 (3.85-4.25)	3.98 (3.76-4.16)	4.30 (4.28-4.39)	4.45 (4.39-4.75)	1.1	0.007
**DPP7**	Q9UHL4	3.34 (2.77-3.77)	3.59 (2.92-4.04)	3.82 (3.59-4.32)	4.96 (4.60-5.28)	7	0.008
**CANT1**	Q8WVQ1	5.36 (5.23-5.63)	5.60 (5.42-5.78)	5.78 (5.57-5.86)	5.91 (4.60-5.28)	0.8	0.01
**ALDH1A1**	P00352	2.66 (2.20-3.52)	3.75 (2.20-4.70)	3.29 (3.14-3.84)	5.13 (3.32-5.77)	10.3	0.019
**TYMP**	P19971	7.24 (6.95-7.40)	7.85 (7.37-8.26)	7.58 (7.37-8.06)	8.33 (7.44-9.16)	3.9	0.019
**ADGRE2**	Q9UHX3	5.39 (5.14-5.67)	5.50 (5.15-5.69)	5.73 (5.60-6.13)	5.85 (5.66-6.16)	1.1	0.02
**KYAT1**	Q16773	6.24 (5.69-6.62)	6.39 (5.97-7.81)	6.36 (6.13-6.80)	7.45 (7.32-8.26)	5.3	0.02
**MCFD2**	Q8NI22	2.38 (2.14-2.51)	2.40 (2.24-2.68)	2.60 (2.43-2.81)	2.86 (3.01-2.67)	0.8	0.02
**SEMA3F**	Q13275	4.05 (3.80-4.19)	4.15 (3.98-4.27)	4.37 (4.19-4.57)	4.52 (4.05-4.96)	1	0.02
**THOP1**	P52888	4.50 (4.19-4.82)	4.74 (4.38-5.36)	4.96 (4.59-5.19)	5.22 (4.86-5.44)	1.6	0.02
**TXNDC5**	Q8NBS9	1.63 (1.39-1.92)	1.87 (1.42-1.94)	2.08 (1.87-2.14)	2.23 (1.96-2.29)	1	0.02
**SSC4D**	Q8WTU2	6.89 (6.24-8.53)	9.02 (5.26-10.67)	11.1 (8.84-11.71)	9.54 (7.45-11.39)	36.7	0.031
**FBP1**	P09467	0.42 (0.06-0.79)	0.67 (0.28-1.11)	0.87 (0.69-1.12)	1.25 (0.32-1.70)	1.8	0.045
**CES1**	P23141	3.55 (3.02-3.79)	3.95 (3.50-5.10)	4.63 (3.82-5.29)	5.67 (4.43-6.29)	13.3	0.031

Expression values are reported as NPX median value (IQR). S0, Steatosis grade 0; S1, Steatosis grade 1; S2, Steatosis grade 2; S3, Steatosis grade 3.

### 3.5 Selection of a circulating protein panel to detect liver steatosis in individuals with PWS

**Figure 4:**
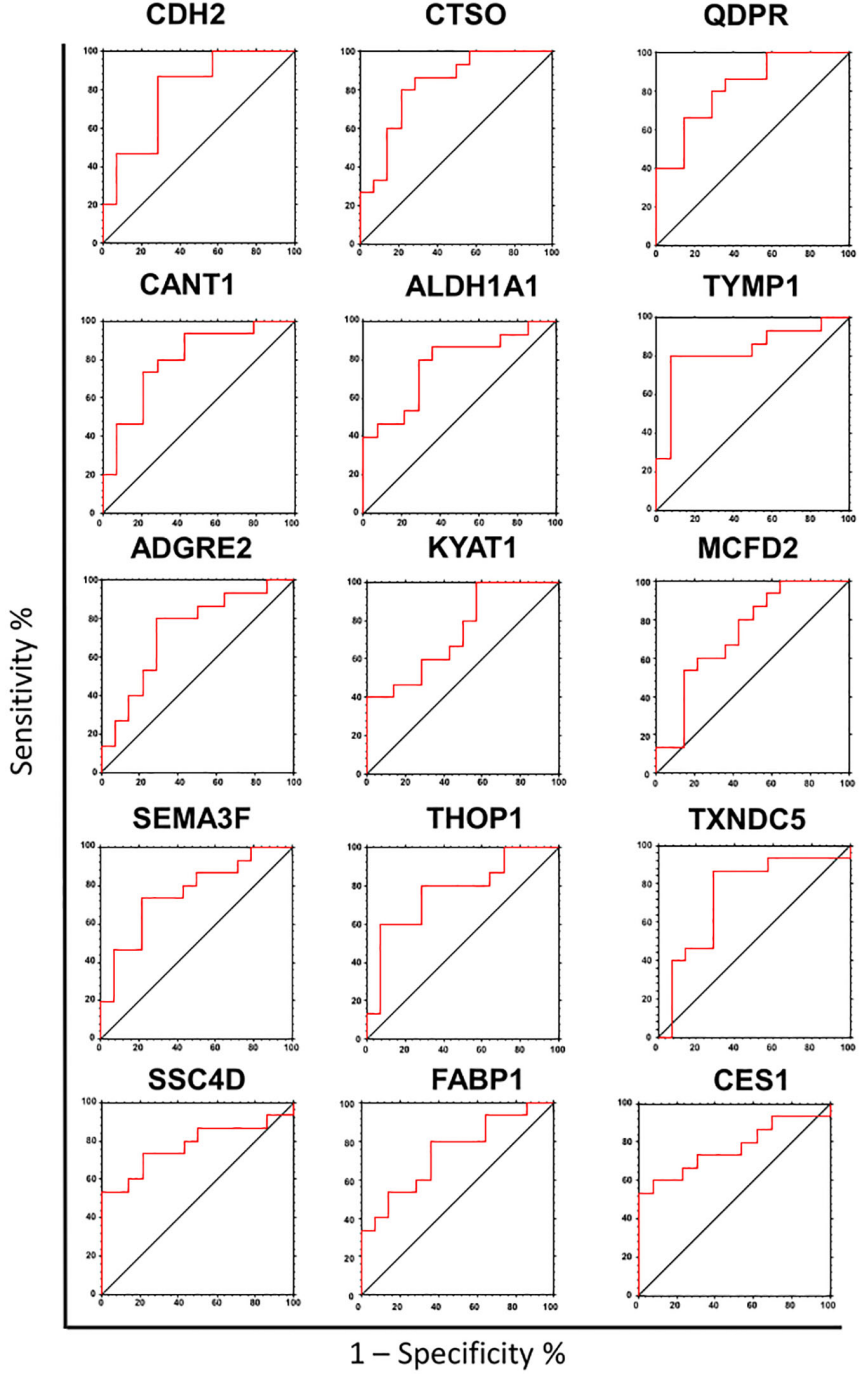
Receiver operating characteristic (ROC) analysis of the biomarker candidates. ROC curve analysis was used to determine the discriminatory potential of candidates according to the presence of liver steatosis.

**Figure 5:**
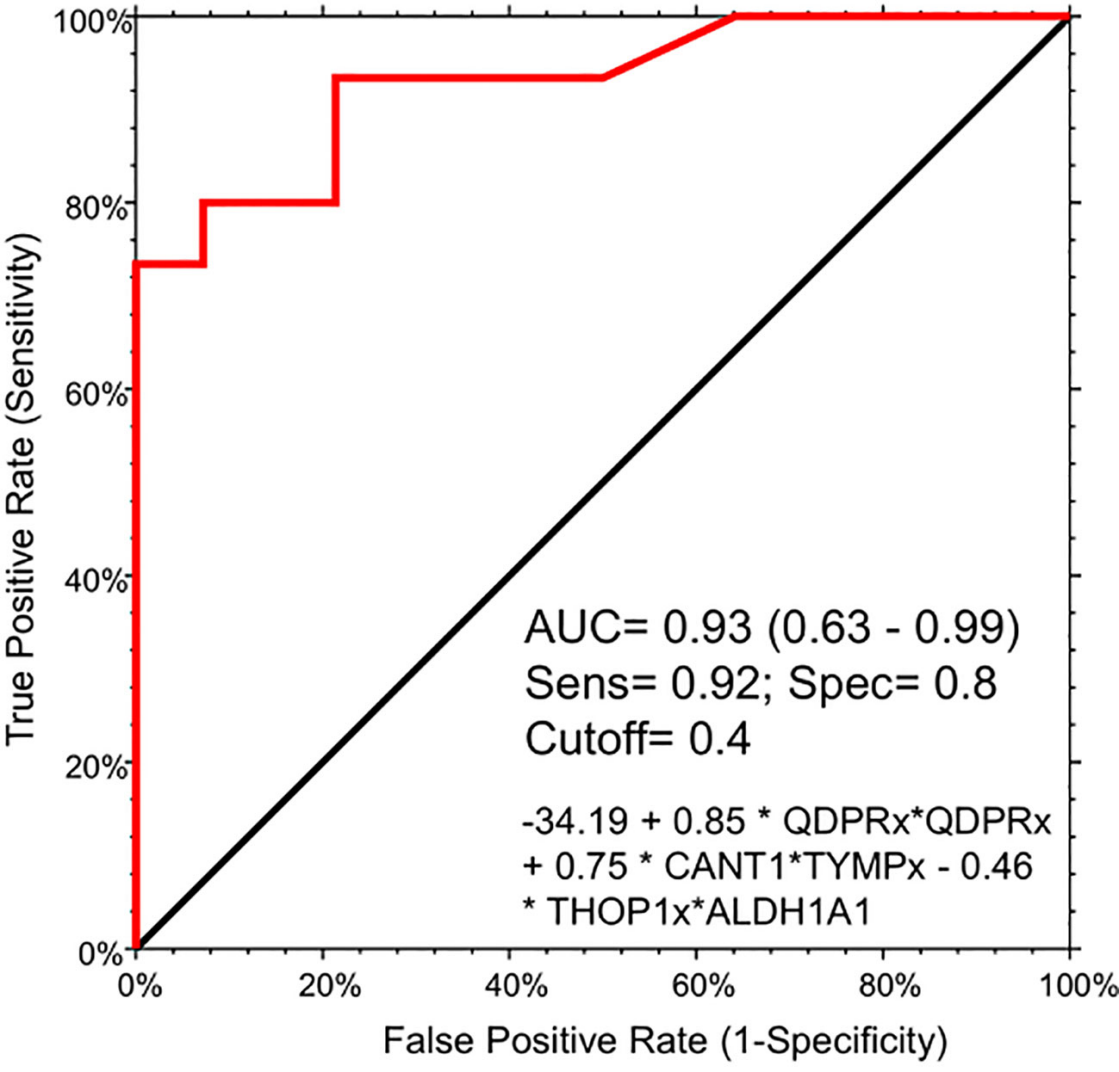
Logistic regression model for the detection of liver steatosis in individuals with PWS. The combination of the five circulating protein biomarkers in the logistic regression model demonstrated an AUC of 0.93 (95% CI: 0.63-0.99), with a sensitivity of 93% and specificity of 80%, at a cut-off value determined at 0.4. The logistic model is the following: -34.19 + 0.85 * QDPR*QDPR + 0.75 * CANT1*TYMP - 0.46 * THOP1*ALDH1A1.

To assess the potential of the significant biomarkers in distinguishing between subjects with or without steatosis, a ROC curve analysis was conducted on the 15 identified biomarkers. The analysis involved determining the AUC (Area Under the Curve) and cut-off values based on the Youden index, which maximizes sensitivity and specificity for each candidate. The results, including the AUC values and cut-off values, are presented in **Table 4** and **Figure 4**. p-values, sensitivity, and specificity values were considered to rank the performance in distinguishing the two groups. The top five candidates were selected for further analysis using logistic regression with forward selection with switching. The resulting model, -34.19 + 0.85 * QDPR*QDPR + 0.75 * CANT1*TYMP - 0.46 * THOP1*ALDH1A1, demonstrated an AUC of 0.93 (95% CI: 0.63-0.99), with a sensitivity of 93% and specificity of 80% at a cut-off value determined at 0.4 (**Figure 5**).

**Table 4:** Ranked list of the AUC values calculated for each of the biomarker candidates.

PROTEIN	UniProt	AUC	95% CI		p-value	cutoff value	(sens.)	(spec.)
**TYMP**	P19971	0.83	0.59	- 0.94	p >0.0001	≥ 7.42	0.80	0.79
**QDPR**	P09417	0.82	0.60	- 0.93	p >0.0001	≥ 3.21	0.87	0.64
**CTSO**	P43234	0.82	0.59	- 0.93	p >0.0001	≥ 2.99	0.87	0.71
**CANT1**	Q8WVQ1	0.80	0.56	- 0.91	0.0003	≥ 5.60	0.80	0.71
**CDH2**	P19022	0.79	0.55	- 0.91	0.0004	≥ 4.15	0.87	0.71
**ALDH1A1**	P00352	0.78	0.53	- 0.90	0.001	≥ 3.09	0.80	0.71
**SSC4D**	Q8WTU2	0.78	0.52	- 0.90	0.0015	≥ 8.91	0.73	0.79
**THOP1**	P52888	0.77	0.52	- 0.90	0.0015	≥ 4.69	0.80	0.71
**CES1**	P23141	0.77	0.52	- 0.90	0.002	≥ 3.78	0.73	0.69
**SEMA3F**	Q13275	0.76	0.52	- 0.89	0.0021	≥ 4.16	0.73	0.79
**KYAT1**	Q16773	0.74	0.50	- 0.88	0.0045	≥ 6.44	0.60	0.71
**FBP1**	P09467	0.74	0.50	- 0.88	0.0046	≥ 0.64	0.80	0.64
**TXNDC5**	Q8NBS9	0.74	0.47	- 0.89	0.0081	≥ 1.85	0.87	0.71
**MCFD2**	Q8NI22	0.73	0.48	- 0.87	0.0083	≥ 2.48	0.67	0.64
**ADGRE2**	Q9UHX3	0.73	0.48	- 0.87	0.0084	≥ 5.59	0.80	0.71

AUC, area under the curve; CI, confidential interval; Sens., sensitivity; Spec., specificity.

Due to the rarity of the disease and the general scarcity of sufficient biological samples for validating the logistic model, we assessed the model’s robustness through a bootstrapping analysis with out-of-bag prediction over 10,000 iterations. This *in silico* simulation provided additional validation for our model. We evaluated the logistic regression model using observations from the bootstrap sample, resulting in an AUC of 0.83, a sensitivity of 0.81 and aspecificity of 0.87 at a determined cut-off of 0.554 (**Figure S2**). Interestingly, the correlation analysis evidenced a high degree of association (r >0.8 and p < 0.001) among the protein biomarkers included in the logistic regression model. Indeed, QDPR highly correlates with KYAT1 (r = 0.89, p < 0.001), ALDH1A1 (r = 0.86, p < 0.001), and THOP1 (r = 0.82, p < 0.001).

**Figure 6:**
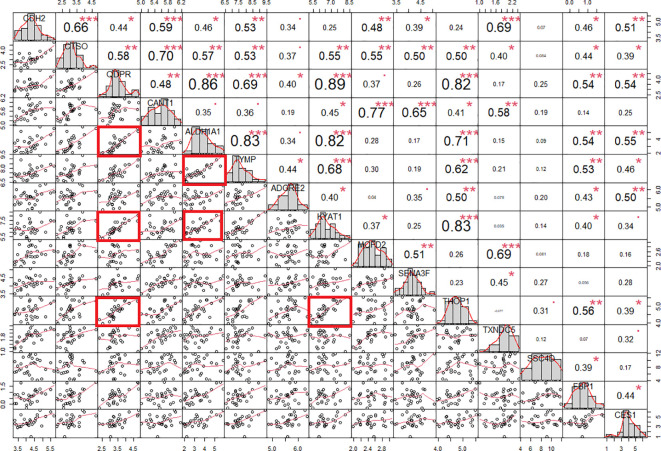
Protein correlation chart in individuals with PWS. The upper-right section displays the correlation coefficient and corresponding p-value for each pairwise correlation, while the lower-left section presents the plots illustrating these correlations. We calculated the correlation coefficient using Spearman’s method. Notably, correlation coefficients exceeding 0.80 with a p-value of less than 0.001 are denoted by red squares. Statistical significance levels are denoted as follows: *, p<0.05; **, p<0.01; ***, p<0.001.

**Figure 7:**
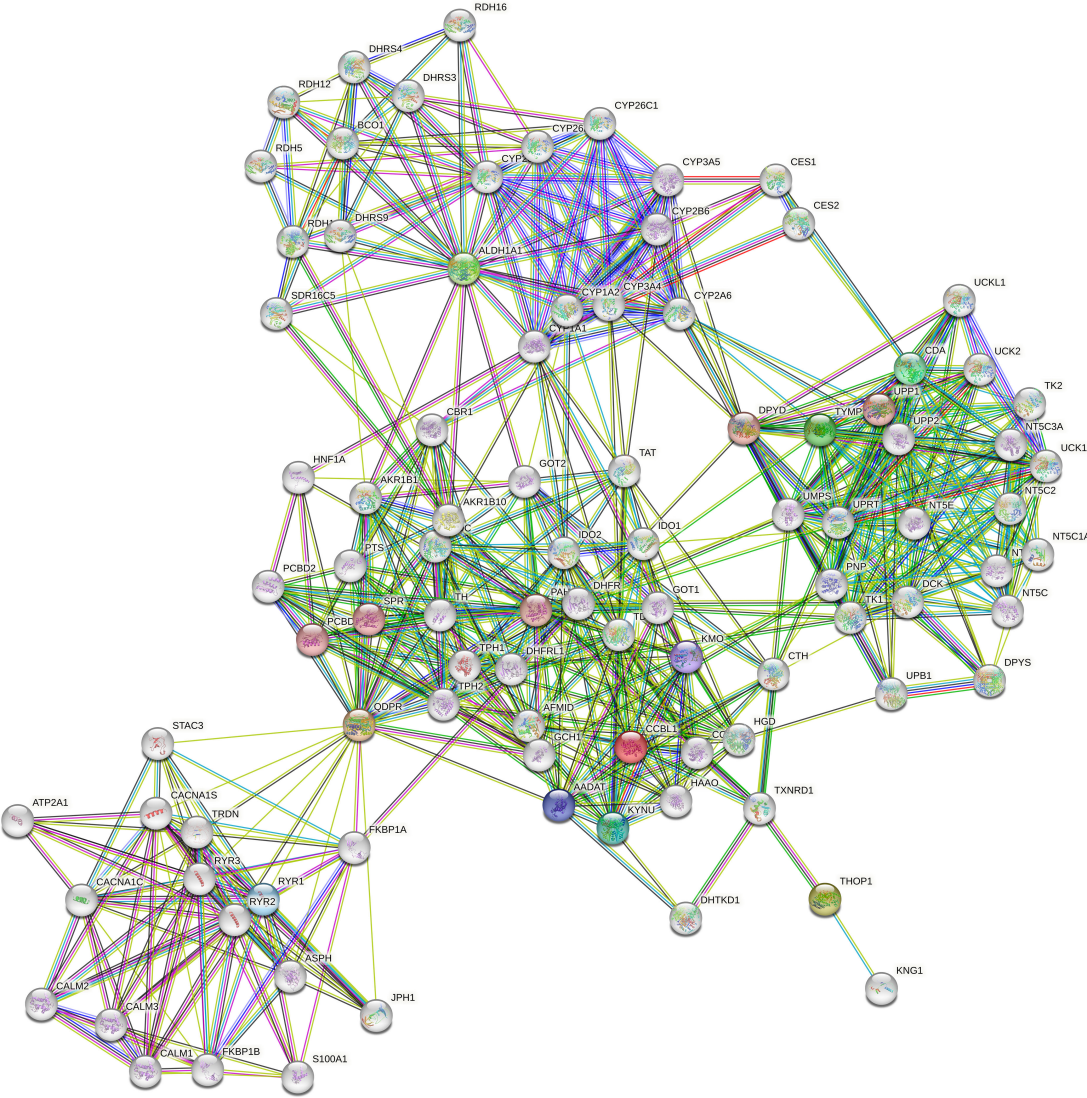
Protein-protein interaction network. The network was constructed in STRING Version 11.5 by using a 5-gene list consisting of QDPR, KYAT1 (also known as CCBL1), ALDH1A1, THOP1 and TYMP. The network consists of 62 nodes encompassing 7 main enriched pathways.

ALDH1A1 correlated with TYMP (r = 0.83, p < 0.001) and KYAT1 (r = 0.82, p < 0.001), also known as CCBL1, which in turn correlated with THOP1 (R2 = 0.83, p>0.001) (**Figure 6**). Consistently, these candidates demonstrated interconnections at different levels of interaction, forming a network of 62 nodes (**Figure 7**) primarily involved in 7 metabolic pathways listed in **Table 5**.

**Table 5:** Enriched panther pathways.

PANTHER Pathways	#Homo sapiens (REF)	Mapped terms	expected	Fold Enrichment	p-value
5-Hydroxytryptamine biosynthesis	3	3	.01	> 100	3.22E-04
Salvage pyrimidine deoxyribonucleotides	4	3	.02	> 100	5.61E-04
Tetrahydrofolate biosynthesis	5	3	.02	> 100	8.95E-04
Pyrimidine Metabolism	11	6	.05	> 100	1.87E-08
Salvage pyrimidine ribonucleotides	13	7	.06	> 100	5.22E-10
Beta2 adrenergic receptor signaling pathway	47	5	.23	22.12	7.54E-04
Beta1 adrenergic receptor signaling pathway	47	5	.23	22.12	7.54E-04

**Figure 8:**
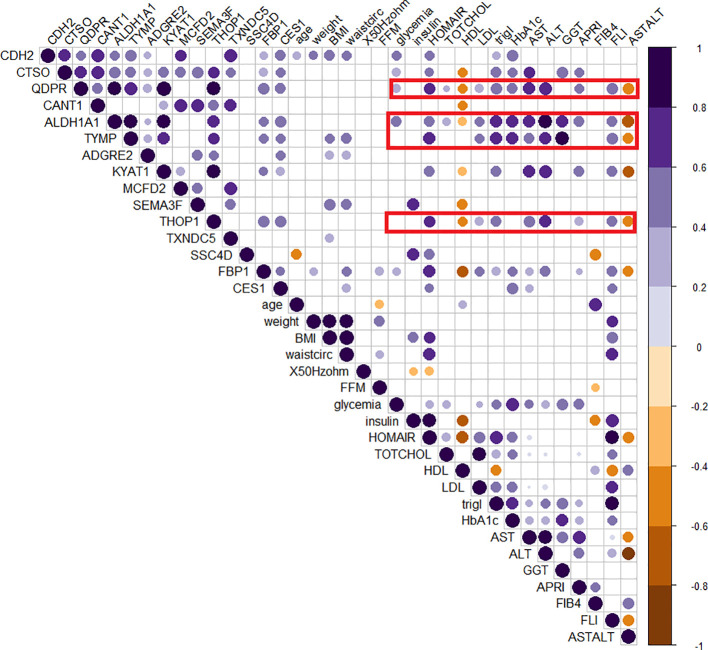
Correlogram of circulating protein candidates and clinical variables. Spearman’s correlation was used to determine the correlation coefficients. The size and color depth of the dots are proportional to the correlation coefficient. Purple colors indicate a positive correlation while brown colors indicate a negative one. Only significant correlations were reported. Red squares highlight the cluster of clinical variables that exhibit correlations with the genes included in the logistic regression model (QDPR, KYAT1, ALDH1A1, THOP1 and TYMP).

**Figure 9:**
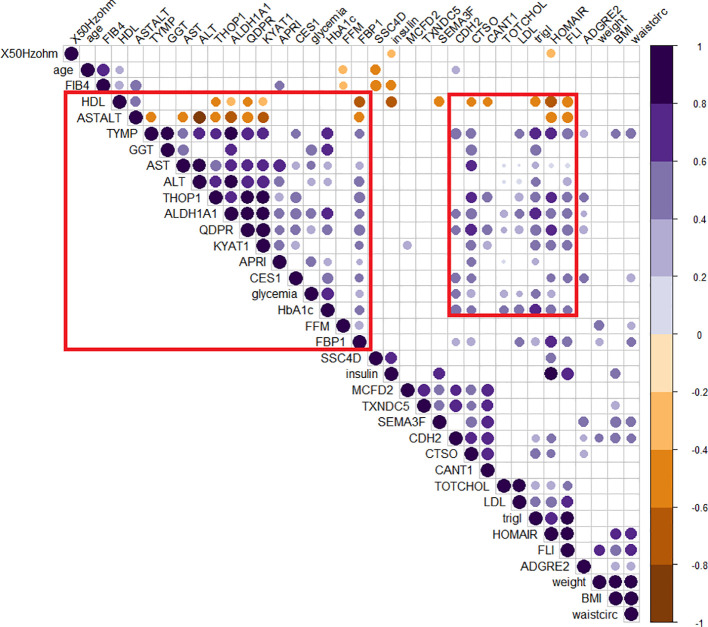
Clusterized correlogram of circulating protein candidates and clinical variables. Spearman’s correlation was used to determine the correlation coefficients. Unsupervised hierarchical clusterization was used to identify similar groups of correlating variables. The size and color depth of the dots are proportional to the correlation coefficient. Purple colors indicate a positive correlation while brown colors are a negative one. Only significant correlations were reported. Red squares highlight clusters of variables associated either with lipid metabolism or liver damage.

The role of those candidates was confirmed by the corrplot matrix, which showed a significant positive correlation of QDPR, ALDH1A1, TYMP, and THOP1 with several clinical variables associated with liver disease, such as cholesterol level, LDL, triglycerides, transaminases levels, and HbA1c, as well as with disease-associated scores such as FLI, APRI, and HOMA (**Figure 8**). A negative correlation was observed between HDL levels and QDPR, ALDH1A1, THOP1, and CANT1. When clustering in the correlation matrix was performed, we identified two interesting clusters, one grouping the protein candidates associated with fat metabolisms-related clinical variables, and the other grouping protein candidates associated with liver injury and risk of diabetes. These clusters, besides the five-protein signature, included also CES1, FBP1, KYAT1, CDH2, and CTSO (**Figure 9**).

## 4 Discussion

The use of biomarkers for detecting and monitoring liver steatosis holds significant potential in individuals with PWS. Liver steatosis, characterized by fat accumulation in the liver cells, is a common complication in PWS and is associated with various metabolic abnormalities and an increased risk of liver-related complications (12, 13, 34).

Circulating biomarkers provide a minimally invasive approach easily obtained from individuals with PWS. This minimally invasive nature of biomarkers not only improves patient comfort and compliance but also allows for repeated measurements, facilitating longitudinal monitoring of liver health and response to therapy. For these reasons, the present study aimed to identify circulating protein biomarkers associated with liver steatosis in individuals with PWS.

Patients with liver steatosis showed a significant increase in BMI, diastolic pressure, insulin, HOMA index, glycated hemoglobin (HbA1c), C-reactive protein, triglycerides, fatty liver index (FLI), and the presence of metabolic syndrome. As for non-syndromic obesity, these findings are consistent with previous studies linking these factors to the development and progression of liver steatosis (35–37).

To further characterize the disease, a circulating proteome analysis was performed.

To the best of our knowledge, our study represents the first attempt to comprehensively investigate the circulating proteome profile in individuals with PWS and its association with steatosis. We identified 15 circulating proteins that exhibited higher expression levels in individuals with PWS and steatosis as compared with those without. These proteins include CDH2, CTSO, QDPR, CANT1, ALDH1A1, TYMP, ADGRE, KYAT1, MCFD2, SEMA3F, THOP1, TXND5, SSC4D, FBP1, and CES1. These proteins have been implicated in various biological processes and metabolic pathways, including those related to liver function.

Upon examining mRNA expression profiles in different organs within the GTEx database, it became evident that the liver exhibited the highest mRNA expression levels of FBP1, CES1, and QDPR, suggesting that these proteins play crucial roles in liver functions. They may be specifically involved in the angiopoietin-like protein 8 regulatory pathway, disorders of fructose metabolism, glycolysis and gluconeogenesis, cholesterol, and sphingolipid transport. Interestingly, the circulating angiopoietin Like 8 (ANGPTL8) has already been associated with liver steatosis in PWS (34).

In particular, serum ANGPTL8 inversely correlated with the severity of liver steatosis and was reported to be usually lower in individuals with PWS than in obese controls (34). Previous reports have highlighted the key role of CES1 in liver lipid metabolism and associated its alterations with obesity, hepatic steatosis, hyperlipidemia, and an increased risk of cardiovascular disease (38–40). CES1 was positively correlated with increased lipid storage and plasma lipid concentration. Furthermore, mutations in CES1, that reduce its lipolytic activity by 80% *in vivo*, have been shown to prevent high-fat diet-induced lipid accumulation in the liver and reduce plasma triacylglycerol levels (41). We did not detect any association between CES1 expression and LDL or triglycerides, however, there was a positive correlation with the FLI index. To further describe the association with the disease, the expression of the circulating proteome was also evaluated in a group of patients with different grades of liver steatosis.

The relevance of 15 biomarkers was highlighted by their distribution according to the extent of fat accumulation, with a progressive increase in the expression of all candidates with steatosis. In addition to those 15 candidates, SIGLEC7 was associated with the progression of steatosis from grade 1 to grade 3 (p< 0.01). It is noteworthy that SIGLEC7 expression in subjects with steatosis grades 2 and 3 was higher compared to subjects without steatosis or with steatosis grade 1. In parallel, DDP7 was higher in the group with grade 3 steatosis suggesting that these candidates perform better in discriminating subjects with more severe steatosis. Both proteins participate in immune system-related pathways. SIGLEC 7 has a sialic acid binding activity involved in cell adhesion and it is expressed in immune cells, while DPP7 is involved in the maintenance of quiescent lymphocytes (42, 43). A recent report showed that SIGLEC7, released in serum from macrophages, was associated with liver fibrosis and was used as a diagnostic biomarker in patients with MAFLD/MASH (44). This can explain why SIGLEC7 level was higher in advanced steatosis where fibrosis is more frequent (45). Similarly, in a proteomic study conducted in a mouse model of Niemann-Pick type C disease (NPCD), an alteration in proteins involved in pathways of liver damage, lipid metabolism, and inflammation was observed, including DPP7 (46, 47), suggesting a possible role of this protein in more advanced stages of liver damage.

In this study, we evaluated the discriminatory potential of the 15 protein candidates in the detection of liver steatosis in individuals with PWS. When a logistic regression model including QDPR, CANT1, TYMP, THOP1, and ALDH1A1 was constructed the model exhibited an AUC of 0.93 (95% CI: 0.63-0.99), with a sensitivity of 93% and specificity of 80%, highlighting the potential of these proteins as biomarkers for the detection of liver steatosis in PWS. Interestingly, these biomarkers well correlated in the serum of individuals with PWS and they are part of a cellular network connecting several metabolic pathways, including 5-Hydroxytryptamine biosynthesis, Salvage pyrimidine deoxyribonucleotides, Tetrahydrofolate biosynthesis, Pyrimidine Metabolism, Salvage pyrimidine ribonucleotides, Beta2 adrenergic receptor signaling pathway, and Beta1 adrenergic receptor signaling pathway.

Consistently, 5-Hydroxytryptamine (5-HT) has been demonstrated to regulate lipid metabolism in the liver through the activation of the mTOR pathway (48, 49). In rats with a high-fat diet or exposed to 5-HT was observed an overproduction of hepatic triglycerides and VLDL determining liver steatosis and hyperlipidemia (50). In our study, we observed a significant positive correlation between the selected 5 protein biomarkers included in the logistic model and various clinical variables associated with liver diseases, such as cholesterol levels, LDL, triglycerides, transaminase levels, and HbA1c. Important to considered is the possible alteration of the 5-HT pathway in individuals with PWS.

By using an imprinting center deletion mouse model for PWS (PWSICdel), Davies and colleagues (2019) demonstrated the abnormal serotonin 2C receptor (5-HT2CR) function in this genetic syndrome (51, 52). Since alterations in the 5-HT pathway may have a role in both neuro-behavioral and lipid metabolism alterations, it would be of particular interest to compare individuals with PWS with a group of non-syndromic patients with obesity to further decipher the role of the selected biomarkers in this particular disease.

Disruption of pyrimidine metabolism was associated with lipid accumulation in the liver (53) and the role of β-adrenergic receptors activation was shown in the increased hepatic lipid accumulation, due to new synthesis or lipogenesis (54, 55). This evidence strengthens our results and sustains the potential role of the selected biomarkers in detecting liver steatosis in these patients.

Our study has some limitations. Firstly, histological confirmation of hepatic steatosis was not performed, as a liver biopsy is invasive, scarcely acceptable by these individuals, and has potential complications. Instead, we relied on non-invasive imaging and blood biomarker assessments as screening tools (56). Secondly, the sample size of our PWS group was relatively small, reflecting the rarity of the syndrome and the challenges in recruiting a larger number of patients. Additionally, our study design was cross-sectional, lacking longitudinal data or follow-up information on the patients. Despite these limitations, our study highlights the significance of circulating proteome profiling in PWS and underscores the potential utility of these identified proteins as valuable biomarkers for the diagnosis and management of steatosis in this patient population. Further validation and replication of our findings are warranted in larger and independent cohorts of individuals with PWS. Additionally, longitudinal-associated studies are needed to investigate the potential of these biomarkers in predicting disease progression and treatment response. Early diagnosis and intervention can help to prevent or slow down the progression of liver disease and associated complications, leading to better clinical outcomes. By regularly monitoring the levels of these biomarkers, healthcare professionals can detect the presence of liver steatosis at an early stage, even before the manifestation of overt symptoms or significant liver damage. Early intervention, such as lifestyle modifications and targeted therapeutic interventions, can then be implemented to mitigate the progression of liver steatosis and associated complications in individuals with PWS.

## Data availability statement

The original contributions presented in the study are included in the article/**Supplementary Material**. Further inquiries can be directed to the corresponding author.

## Ethics statement

The studies involving humans were approved by ethics Committee of Istituto Auxologico Italiano Milan, Italy (ethical committee code: CE: 2022_03_15_07; research code: 01C216; acronym: PROTEOMARKER. The studies were conducted in accordance with the local legislation and institutional requirements. The participants provided their written informed consent to participate in this study.

## Author contributions

DP: Conceptualization, Data curation, Formal Analysis, Investigation, Methodology, Supervision, Writing – original draft, Writing – review & editing. PG: Conceptualization, Data curation, Formal Analysis, Investigation, Methodology, Supervision, Writing – original draft, Writing – review & editing. CB: Conceptualization, Investigation, Methodology, Writing – original draft, Writing – review & editing. SG: Conceptualization, Investigation, Methodology, Writing – review & editing. CT: Conceptualization, Supervision, Writing – original draft, Writing – review & editing. AB: Data curation, Investigation, Methodology, Writing – review & editing. DC: Data curation, Investigation, Methodology, Writing – review & editing. AM: Investigation, Methodology, Writing – review & editing. GG: Data curation, Investigation, Methodology, Writing – review & editing. AS: Conceptualization, Funding acquisition, Investigation, Project administration, Resources, Supervision, Writing – original draft, Writing – review & editing.

## Supplementary material

Supplementary Figure 1 Expression of circulating biomarkers in different human tissues. The heatmap was generated in the GTEx data portal reporting the expression levels of the biomarker candidates in different tissues. Deep blue colors indicate higher mRNA expression levels. While light yellow colors indicate lower mRNA expression levels. Unsupervised hierarchical clustering was used to order genes and tissues.

Supplementary Figure 2 Receiver operating characteristic (ROC) analysis for the selected logistic model. The bootstrap analysis with out of bag prediction was used to validate the logistic regression model (-34.19 + 0.85 * QDPR*QDPR + 0.75 * CANT1*TYMP - 0.46 * THOP1*ALDH1A1.) in a synthetic dataset. The logistic model was able to discriminate the presence of steatosis with an AUC of 0.83, with a sensitivity of 0.81 and a specificity of 0.87.
