# Supplementary material for: Proteome profiling identifies circulating biomarkers associated with hepatic steatosis in subjects with Prader-Willi syndrome
Source: Front Endocrinol (Lausanne). 2023 Nov 15;14:1254778. doi: 10.3389/fendo.2023.1254778 (PMC10684934; doi:10.3389/fendo.2023.1254778)
Supplement: Supplementary file 2 [file Table_2.docx]

**Table S2.** List of the pathways in which CES1, FBP1, and QDPR are involved

| **PROTEIN** | **Pathway** | **Source** |
| --- | --- | --- |
| CES1 | [Aspirin ADME](http://www.reactome.org/PathwayBrowser/#/R-HSA-9749641) | Reactome |
| CES1 | [Biological oxidations](http://www.reactome.org/PathwayBrowser/#/R-HSA-211859) | Reactome |
| CES1 | [Cardiac conduction](http://www.reactome.org/PathwayBrowser/#/R-HSA-5576891) | Reactome |
| CES1 | [Drug ADME](http://www.reactome.org/PathwayBrowser/#/R-HSA-9748784) | Reactome |
| CES1 | Drug metabolism - other enzymes | KEGG |
| CES1 | [Clopidogrel Pathway, Pharmacokinetics](http://www.pharmgkb.org/pathway/PA154424674) | PharmaGKB |
| CES1 | [Fluoropyrimidine Pathway, Pharmacokinetics](http://www.pharmgkb.org/pathway/PA150653776) | PharmaGKB |
| CES1 | [Irinotecan Pathway, Pharmacodynamics](http://www.pharmgkb.org/pathway/PA2029) | PharmaGKB |
| CES1 | [Irinotecan Pathway, Pharmacokinetics](http://www.pharmgkb.org/pathway/PA2001) | PharmaGKB |
| CES1 | [Lidocaine Pathway, Pharmacokinetics](http://www.pharmgkb.org/pathway/PA166182313) | PharmaGKB |
| CES1 | [Fluoropyrimidine activity](https://www.wikipathways.org/index.php/Pathway:WP1601) | wikipathways |
| CES1 | [Heroin metabolism](https://www.wikipathways.org/index.php/Pathway:WP2645) | wikipathways |
| CES1 | [NRF2 pathway](https://www.wikipathways.org/index.php/Pathway:WP2884) | wikipathways |
| CES1 | [Nuclear receptors meta-pathway](https://www.wikipathways.org/index.php/Pathway:WP2882) | wikipathways |
| FBP1 | [Gluconeogenesis](http://www.reactome.org/PathwayBrowser/#/R-HSA-70263) | Reactome |
| FBP1 | [Glucose metabolism](http://www.reactome.org/PathwayBrowser/#/R-HSA-70326) | Reactome |
| FBP1 | [Metabolism of carbohydrates](http://www.reactome.org/PathwayBrowser/#/R-HSA-71387) | Reactome |
| FBP1 | Glucagon signaling pathway | KEGG |
| FBP1 | [gluconeogenesis](https://pubchem.ncbi.nlm.nih.gov/pathway/HUMANCYC:PWY66-399#section=Diagram) | PubChem |
| FBP1 | [gluconeogenesis III](https://pubchem.ncbi.nlm.nih.gov/pathway/METACYC:PWY66-399#section=Diagram) | PubChem |
| FBP1 | [Angiopoietin-like protein 8 regulatory pathway](https://www.wikipathways.org/index.php/Pathway:WP3915) | wikipathways |
| FBP1 | [Disorders of fructose metabolism](https://www.wikipathways.org/index.php/Pathway:WP5178) | wikipathways |
| QDPR | [Metabolism of amino acids and derivatives](http://www.reactome.org/PathwayBrowser/#/R-HSA-71291) | Reactome |
| QDPR | [Phenylalanine and tyrosine metabolism](http://www.reactome.org/PathwayBrowser/#/R-HSA-8963691) | Reactome |
| QDPR | [Phenylalanine metabolism](http://www.reactome.org/PathwayBrowser/#/R-HSA-8964208) | Reactome |
| QDPR | Folate biosynthesis | KEGG |
| QDPR | [L-phenylalanine degradation I (aerobic)](https://pubchem.ncbi.nlm.nih.gov/pathway/METACYC:PHENYLALANINE-DEG1-PWY#section=Diagram) | PubChem |
| QDPR | [phenylalanine degradation/tyrosine biosynthesis](https://pubchem.ncbi.nlm.nih.gov/pathway/HUMANCYC:PHENYLALANINE-DEG1-PWY#section=Diagram) | PubChem |
| QDPR | [Disorders of folate metabolism and transport](https://www.wikipathways.org/index.php/Pathway:WP4259) | wikipathways |
