# Supplementary material for: Proteome profiling identifies circulating biomarkers associated with hepatic steatosis in subjects with Prader-Willi syndrome
Source: Front Endocrinol (Lausanne). 2023 Nov 15;14:1254778. doi: 10.3389/fendo.2023.1254778 (PMC10684934; doi:10.3389/fendo.2023.1254778)
Supplement: Supplementary file 3 [file Table_3.docx]

SUPPLEMENTARY MATERIAL

The proteome profiling identifies circulating biomarkers associated with the presence of hepatic steatosis in subjects with Prader-Willi Syndrome

D. Pascut^1(†)^, PJ. Giraudi^2(†)^, C. Banfi^3^, S. Ghilardi^3^, C. Tiribelli^1,2^, A. Bondesan^4^, D. Caroli^4^, A. Minocci^5^, G. Grugni^4,6^, A. Sartorio^4^

^1^Fondazione Italiana Fegato - ONLUS, Liver Cancer Unit, Trieste, Italy

^2^Fondazione Italiana Fegato - ONLUS, Metabolic Liver Disease Unit, Trieste, Italy

^3^Unit of Functional Proteomics, Metabolomics, and Network analysis, Centro Cardiologico Monzino, IRCCS, Milan, Italy

^4^Istituto Auxologico Italiano IRCCS, Experimental Laboratory for Auxo-endocrinological Research, Piancavallo-Verbania, Italy

^5^Istituto Auxologico Italiano IRCCS, Division of Metabolic Diseases, Piancavallo-Verbania, Italy

^6^Istituto Auxologico Italiano IRCCS, Division of Auxology, Piancavallo-Verbania, Italy

†These authors contributed equally to this work and share the first authorship

**R scrip for Boostrapping with out of bag correction procedure**

data <- data.frame(Outcome = c(), THOP1 = c(),TYMP = c(),ALDH1A1 = c(),QDPR = c(),CANT1 = c())

data$Predictor <- -34.19 + (0.85 * data$QDPR^2) + (0.75 * data$CANT1 * data$TYMP) - (0.46 * data$THOP1 * data$ALDH1A1)

formula <- as.formula("Outcome ~ Predictor")

ctrl <- trainControl(method = "none")

oob_preds <- rep(NA, nrow(data))

true_labels <- rep(NA, nrow(data))

n_bootstraps <- 10000

set.seed(123)

for (i in 1:n_bootstraps) {

bootstrap_indices <- sample(1:nrow(data), replace = TRUE)

bootstrap_data <- data[bootstrap_indices, ]

model <- glm(formula, data = bootstrap_data, family = "binomial", control = glm.control(maxit = 1000, epsilon = 1e-6))

oob_indices <- setdiff(1:nrow(data), bootstrap_indices)

oob_preds[oob_indices] <- predict(model, newdata = data[oob_indices, ], type = "response")

true_labels[oob_indices] <- data$Outcome[oob_indices]

}

true_labels <- data$Outcome

roc_obj <- roc(true_labels, oob_preds)

roc_auc <- auc(roc_obj)

sensitivity <- sensitivity(roc_obj, "best")

specificity <- specificity(roc_obj, "best")

cat("OOB AUC:", roc_auc, "\n")

cat("Sensitivity:", sensitivity, "\n")

cat("Specificity:", specificity, "\n")

plot.roc(roc_obj)
