# Supplementary figures and images for: Proteome profiling identifies circulating biomarkers associated with hepatic steatosis in subjects with Prader-Willi syndrome
Source: Front Endocrinol (Lausanne). 2023 Nov 15;14:1254778. doi: 10.3389/fendo.2023.1254778 (PMC10684934; doi:10.3389/fendo.2023.1254778)

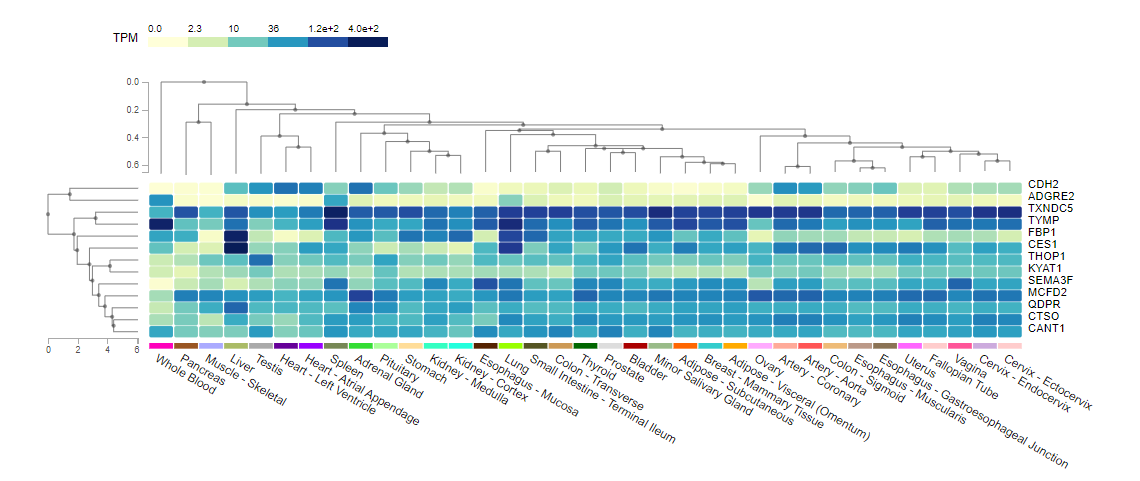

Supplement: Supplementary Figure 1 — Expression of circulating biomarkers in different human tissues. The heatmap was generated in the GTEx data portal reporting the expression levels of the biomarker candidates in different tissues. Deep blue colors indicate higher mRNA expression levels. While light yellow colors indicate lower mRNA expression levels. Unsupervised hierarchical clustering was used to order genes and tissues. [file Image_1.tif]

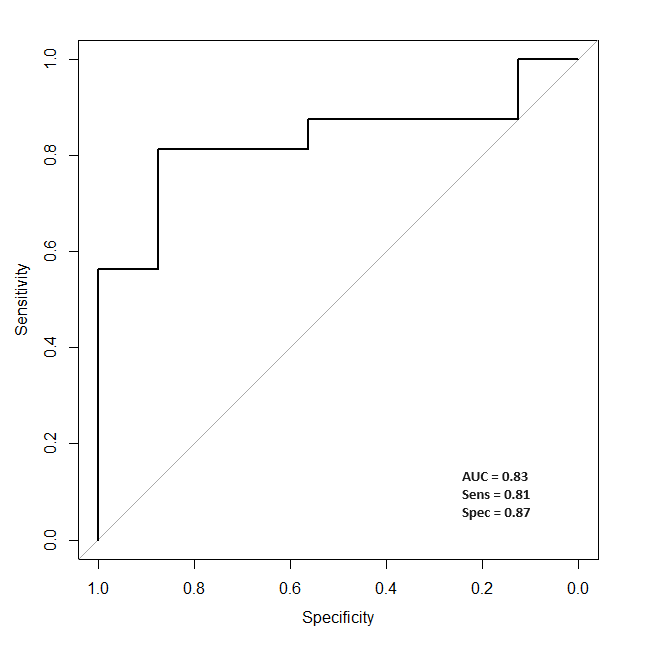

Supplement: Supplementary Figure 2 — Receiver operating characteristic (ROC) analysis for the selected logistic model. The bootstrap analysis with out of bag prediction was used to validate the logistic regression model (-34.19 + 0.85 * QDPR*QDPR + 0.75 * CANT1*TYMP - 0.46 * THOP1*ALDH1A1.) in a synthetic dataset. The logistic model was able to discriminate the presence of steatosis with an AUC of 0.83, with a sensitivity of 0.81 and a specificity of 0.87. [file Image_2.tif]
